# Synthesis of Compounds of the Pyrimidine Series Based on the Reactions of 3-Arylmethylidenefuran-2(3*H*)-ones with *N*,*N*-Binucleophilic Reagents

**DOI:** 10.3390/molecules22081251

**Published:** 2017-07-28

**Authors:** Tatyana Aniskova, Vyacheslav Grinev, Alevtina Yegorova

**Affiliations:** 1 Institute of Chemistry, Chernyshevsky Saratov State University, Ulitsa Astrakhanskaya 83, 410012 Saratov, Russia; grinev@ibppm.ru (V.G.); yegorovaay@gmail.com (A.Ye.); 2 Institute of Biochemistry and Physiology of Plants and Microorganisms RAS, Prospekt Entuziastov 13, 410049 Saratov, Russia

**Keywords:** pyrimidine derivatives, dihydrofuro[2,3-*d*]pyrimidines, pyrimidinylmethylene-phenylfuran-2(3*H*)-ones, biological active compounds, plant-growth regulators, 5-*R*-3-arylmethylidenefuran-2(3*H*)-ones, binucleophilic reagents, heterocyclization

## Abstract

The arylmethylidene derivatives of furan-2(3*H*)-ones are important building blocks for the synthesis of various heterocyclic compounds containing pyrimidine and pyridazine structural fragments, analogues of nitrogen-containing bases of pyrimidine series. In order to continue the development of constructing of molecules containing pyridine and pyridazine fragments, this article is devoted to the synthesis of new biologically active compounds with these moieties. The introduction of a heterocyclic chromenone fragment changes the previously observed 5-*R*-3-arylmethylidenefuran-2(3*H*)-ones route of reaction with guanidine carbonate and leads to 3-[(2-amino-4-(2-hydroxyphenyl)pyrimidin-5-yl)methylene]-5-phenylfuran-2(3*H*)-ones (**2a**–**d**). The structure of the reaction products depends on the nature of the aromatic substituent at the C-3 position of the furanone ring. The interaction of 5-aryl-3-arylmethylidenefuran-2(3*H*)-ones (**1e**–**h**) with thiourea in the basic medium leads to the isolation of 5-(2-oxo-2-phenylethyl)-6-aryl-2-thioxotetrahydropyrimidine-4(1*H*)-ones (**3a**–**d**), demonstrating pronounced plant-growth regulatory activity. Optimal conditions for all discussed processes were developed.

## 1. Introduction

Heterocyclic compounds containing a pyrimidine fragment can be natural or synthetic analogues of nucleosides and nucleotides. They are of considerable interest due to a variety of their biological activities. The pyrimidine structural moiety is responsible for many kinds of biological activity, and is found in the molecules of natural compounds (guanidine, folic acid, etc.) as well as synthetic drugs (antitumor, antiviral, antibacterial drugs, etc.) [1,2,3,4,5,6,7,8].

**Figure 1:**
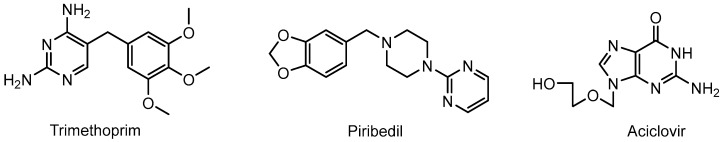
Structures of some medicines containing a pyrimidine moiety.

In the market, there are some approved, effective, well-known medicines containing pyrimidine moieties (Figure 1), such as antimicrobial agent Trimethoprim [9], antiparkinsonian agent Piribedil [10], and antiviral medication Aciclovir (Zovirax) [11], etc. Currently, the interest in pyrimidin-containing biologically active compounds is still significant [12,13,14].

Synthesis of six-membered diazaheterocycles, including their fused analogues, is most often based on the use of binucleophiles containing C=N and C-NH_2_ structural fragments such as guanidine, substituted guanidines, and thiourea. Guanidine compounds are widely abundant in nature. Guanidine moieties serve as active beginnings of many widely used drugs, including antibiotics. The derivatives of guanidine possess bactericidal and fungicidal activity.

Chemically, guanidine and thiourea are *N*,*N*-binucleophiles, which are characterized by high reactivity, allowing one to use them as reagents for the formation of new nitrogen-containing heterocyclic systems with two nitrogen atoms. Guanidine is a physiologically active compound that plays an important role in the metabolism process. Generally, heterocyclization proceeds with the participation of the N–C–N fragment of guanidine. The choice of the substrate for the reactions with those binucleophiles is crucial.

As a substrate, 3-arylmethylidenefuran-2(3*H*)-ones have some advantages. They can be easily obtained, and they are convenient, cost-effective substrates for the synthesis of various hard-to-reach heterocyclic, spirocyclic, and polycyclic compounds [15,16,17,18,19,20,21,22,23,24,25]. 3-Arylmethylidenefuran-2(3*H*)-ones are substances which combine the properties of internal esters and α,β-unsaturated carbonyl compounds, and they are able to react with substances with mobile hydrogen atoms. From this point of view, the arylmethylidene derivatives of furan-2(3*H*)-ones are suitable building blocks for the synthesis of various heterocyclic compounds containing pyrimidine and pyridazine structural fragments. These fragments are the moieties of nucleic acids and coenzymes. Also, they are responsible for the transfer and storage of hereditary information. In connection with this, the development of new synthetic methods for the construction of molecules containing pyridine and pyridazine fragments is a promising direction in the organic synthesis of biologically active compounds.

**Scheme 1:**
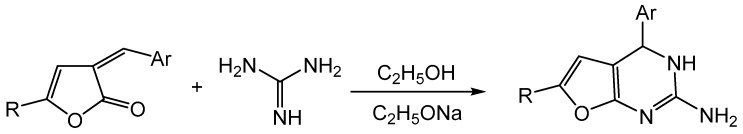
Synthesis of 4-Ar-6-*R*-3,4-dihydrofuro[2,3-*d*]pyrimidine-2-amines.

The interaction of guanidine carbonate with 5-*R*-3-arylmethylidenefuran-2(3*H*)-one has been previously studied [26]. As a result of this reaction, the isolated products, on the basis of the physicochemical methods, were characterized as 4-Ar-6-*R*-3,4-dihydrofuro[2,3-*d*]pyrimidine-2-amines (Scheme 1).

Taking into account the high reactivity of guanidine and the presence of several electrophilic centers in the arylmethylidene derivatives of furan-2(3*H*)-ones, several directions of the reaction can be expected. However, the structure of 4-Ar-6-*R*-3,4-dihydrofuro[2,3-*d*]pyrimidine-2-amines allows us to suggest the conversion scheme, which includes an initial attack of the guanidine amino group on the carbonyl moiety of furan-2-one, followed by the opening of the lactone ring. The stabilization of the formed intermediate occurs due to the attack of the imino group on a double bond C=C, followed by the enolyzation and dehydration under the action of hydrochloric acid.

## 2. Results and Discussion

The synthetic potential of this reaction can be significantly broadened by the presence of a chromenone fragment in the structure of furan-2(3*H*)-ones. 3-[(2-Oxo-5-phenylfuran-3-ylidene)methyl]-4*H*-chromen-4-ones (**1a**–**d**) are promising polyfunctional compounds containing several non-equivalent reaction centers, which makes them attractive substrates for reactions with nucleophilic reagents. In this case, the routes of these transformations depend on the chosen nucleophilic reagent, as well as on the reaction conditions.

**Scheme 2:**
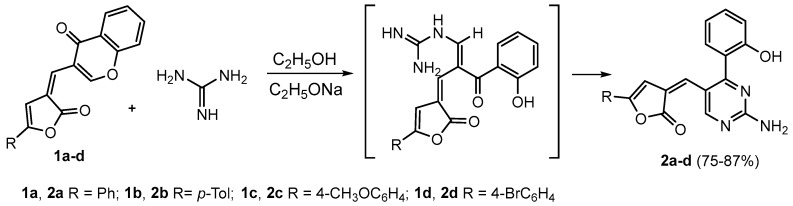
Synthesis of 3-[(2-amino-4-(2-hydroxyphenyl)pyrimidin-5-yl)methylene]-5-phenylfuran-2(3*H*)-ones (**2a**–**d**).

The reaction of substituted furan-2-ones (**1a**–**d**) with guanidine was studied for the construction of the pyrimidine ring linearly bound to the furan-2-one fragment. The optimum process conditions are the boiling of the reagent in ethanol for 5 h in the presence of sodium ethoxide (Scheme 2). The introduction of the heterocyclic chromenone fragment changes the route of reaction and affects the time of the process and the yields of the products.

As a result, the reaction products, stable pyrimidine structures of 3-[(2-amino-4-(2-hydroxyphenyl)pyrimidin-5-yl)methylene]-5-phenylfuran-2(3*H*)-ones (**2a**–**d**), were isolated.

The ^1^H-NMR spectra of the newly synthesized compounds (**2a**–**d**) contain the broadened singlet of the hydroxyl group at 1.35–1.43 ppm, the furan ring singlet at 6.56–6.78 ppm, the proton singlet of the multiple exocyclic C=C bond at 7.05–7.13 ppm, the singlet of the protons of the amino group at 8.25–8.37 ppm, and the proton singlet of the pyrimidine ring at 9.97–10.12 ppm. In the ^13^C-NMR spectra, the most characteristic observations include the signal of the carbon atom of the carbonyl group at 166.3–169.5 ppm, the signal of the carbon atom of the C-4 pyrimidine ring at 156.2–158.4 ppm, the signal of the C-2 carbon atom of the pyrimidine ring at 161.8–164.4 ppm, a carbon atom signal of the OH group at 159.1–160.9 ppm, and a series of signals of *sp*^2^-hybridyzed carbon atoms at 101.3–139.8 ppm.

Under these conditions, the initial attack of the amino group of guanidine is probably directed to the C-2 atom of the chromenone cycle, which leads to the disclosure of the latter ring. The further attack of the imino group on the carbonyl group results in the formation of a substituted stable pyrimidine system. Other probable directions of the reaction are not implemented.

For the purpose of synthesizing promising plant-growth regulating agents [27] containing a pyrimidine moiety in their structure, another cyclizing agent, thiourea, was used. Thiourea interacts with compounds of various classes that contain electrophilic centers in their structure. Depending on the catalyst used, thiourea can react at different centers, with the formation of heterocyclic compounds of different structures. The interaction between arylmethylidene derivatives of furan-2-ones (**1e**–**h**) and thiourea was studied by refluxing in the basic medium.

**Scheme 3:**
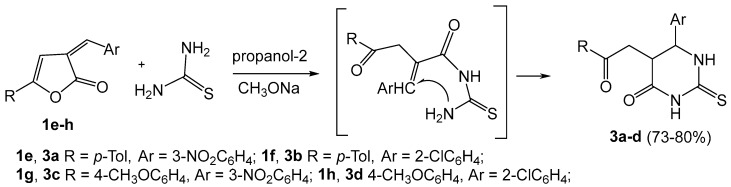
Synthesis of 5-(2-oxo-2-phenylethyl)-6-aryl-2-thioxotetrophydropyrimidine-4(1*H*)-ones (**3a**–**d**).

The reaction of 5-aryl-3-arylmethylidenefuran-2(3*H*)-ones (**1e**–**h**) with thiourea at a ratio of 1:1.5 with heating for 5 h in isopropyl alcohol with a catalytic amount of sodium methoxide led to the isolation of products which, according to elemental analysis and spectral characteristics, correspond to 5-(2-oxo-2-phenylethyl)-6-aryl-2-thioxotetrophydropyrimidine-4(1*H*)-ones (**3a**–**d**) with a yield of up to 76% (Scheme 3).

In the ^1^H-NMR spectra of compounds **3a**–**d**, the signals of the protons of the NH group in the regions of 1.24–1.56 ppm and 4.18–4.52 ppm were noted, as well as the doublet of the proton of the tertiary carbon atom at 4.82–5.27 ppm, the multiplet of the proton of the tertiary carbon atom at 2.95–3.35 ppm, two doublets of protons at the secondary carbon atom in the regions of 3.25–3.78 ppm and 1.87–2.90 ppm, and a series of signals of aromatic protons in a weak field at 6.84–8.01 ppm.

Under the basic catalysis conditions, the direction of attack of the nucleophilic center is as follows. The amino group of thiourea attacks the carbon atom of the carbonyl group, which has a maximum electron density deficit that leads to the opening of the lactone cycle and passes through the stage of formation of the substituted amide of the substituted ketoacid. However, it was not possible to isolate the noncyclic intermediate. The cyclization of the intermediate under conditions of basic catalysis goes through the nitrogen atom of the second amino group of thiourea to form a stable pyrimidine ring in compounds **3a**–**d**.

## 3. Materials and Methods

### 3.1. General

The ^1^H- and ^13^C-NMR spectra were recorded at 20–25 °C on a Varian-400 spectrometer (400 and 100 MHz, respectively; Agilent Technologies, Santa Clara, CA, USA), using CDCl_3_ as a solvent and tetramethylsilane as an internal standard. Analytical thin layer chromatography TLC was performed using Silufol UV-254 plates (hexane-ethyl acetate-chloroform, 2:2:1; development with iodine vapor). The melting points were measured in open capillaries. The elemental analyses were obtained on a Vario Micro cube Elementar CHNS analyzer (Elementar Analysensysteme GmbH, Hanau, Germany). 3-[(2-Oxo-5-phenylfuran-3-ylidene)methyl]-4*H*-chromen-4-ones (**1a**–**d**) as well as 5-aryl-3-arylmethylidenefuran-2(3*H*)-ones (**1e**–**h**) were synthesized according to References [22,28], respectively.

### 3.2. Synthesis of Compounds ***2a**–**d***

A mixture of 0.01 moles of 5-R-furan-2(3*H*)-one (**1a**–**d**) and 0.01 moles of guanidine carbonate was refluxed in 15 mL of ethanol for 5 h in the presence of 0.001 moles of sodium ethoxide. The cooled mixture was treated with concentrated hydrochloric acid to achieve a neutral pH. The precipitated crystals were filtered off, and then washed with water. The resulting crystals were recrystallized from propanol-2.

*3-[(2-Amino-4-(2-hydroxyphenyl)pyrimidin-5-yl)methylene]-5-phenylfuran-2(3H)-one* (**2a**). Yield: 78%. m.p.: 199–201 °C. ^1^H-NMR (400 MHz, CDCl_3_) δ 1.35 (s, 1H, OH), 6.59 (s, 1H, Fur), 7.05 (s, 1H, CH-Ar), 8.26 (s, 2H, NH_2_), 10.05 (s, 1H, C-6 pyrimidine), 7.22–7.57 (m, 9H, Ar). ^13^C-NMR (100 MHz, CDCl_3_) δ 102.4, 112.7, 114.2, 116.8, 120.3, 121.6, 122.7, 123.1, 123.9, 124.2, 125.8, 125.9, 126.3, 127.7, 129.5, 130.1, 132.7, 157.3 (C-4 pyrimidine), 159.9 (C-OH), 161.9 (C-2 pyrimidine), 166.3 (C=O), Anal. calcd. for C_21_H_15_N_3_O_3_: C 70.58, H 4.23, N 11.76; found: C 70.98, H 3.78, N 11.83.

*3-[(2-Amino-4-(2-hydroxyphenyl)pyrimidin-5-yl)methylene]-5-(p-tolyl)furan-2(3H)-one* (**2b**). Yield: 82%. m.p.: 213–215 °C. ^1^H-NMR (400 MHz, CDCl_3_) δ 1.39 (s, 1H, OH), 2.35 (s, 3H, СН_3_), 6.56 (s, 1H, Fur), 7.11 (s, 1H, CH-Ar), 8.29 (s, 2H, NH_2_), 9.97 (s, 1H, C-6 pyrimidine), 7.24 (d, 2Н, *J* = 8.1 Hz, *p*-Tol), 7.41 (d, 2Н, *J* = 8.1 Hz, *p*-Tol), 7.28–7.39 (m, 4H, Ar). ^13^C-NMR (100 MHz, CDCl_3_) δ 25.3, 101.3, 109.1, 116.0, 117.2, 121.2, 123.7 124.2, 125.0, 125.6, 128.2, 129.3, 131.3, 132.2, 134.8, 136.9, 156.3 (C-4 pyrimidine), 159.1 (C-OH), 162.2 (C-2 pyrimidine), 167.6 (C=O), Anal. calcd. for C_22_H_17_N_3_O_3_: C 71.15, H 4.61, N 11.31; found: C 71.62, H 4.93, N 10.95.

*3-[(2-Amino-4-(2-hydroxyphenyl)pyrimidin-5-yl)methylene]-5-(4-methoxyphenyl)-furan-2(3H)-one* (**2c**). Yield: 87%. m.p.: 219–221 °C. ^1^H-NMR (400 MHz, CDCl_3_) δ 1.37 (s, 1H, OH), 3.55 (s, 3H, ОСН_3_), 6.61 (s, 1H, Fur), 7.08 (s, 1H, CH-Ar), 8.30 (s, 2H, NH_2_), 10.03 (s, 1H, C-6 pyrimidine), 7.29 (d, 2Н, *J* = 8.1 Hz, Ar), 7.36 (d, 2Н, *J* = 8.1 Hz, Ar), 7.26–7.32 (m, 4H, Ar). ^13^C-NMR (100 MHz, CDCl_3_) δ 58.2, 103.1, 107.4, 111.6, 115.0, 115.4, 119.3 123.7, 128.2, 131.1, 132.7, 133.1, 134.8, 135.1, 135.7, 136.1, 158.4 (C-4 pyrimidine), 160.9 (C-OH), 164.4 (C-2 pyrimidine), 168.2 (C=O), Anal. calcd. for C_22_H_17_N_3_O_4_: C 68.21, H 4.42, N 10.85; found: C 68.54, H 4.15, N 11.17.

*3-[(2-Amino-4-(2-hydroxyphenyl)pyrimidin-5-yl)methylene]-5-(4-bromophenyl)-furan-2(3H)-one* (**2d**). Yield: 75%. m.p.: 186–188 °C. ^1^H-NMR (400 MHz, CDCl_3_) δ 1.43 (s, 1H, OH), 6.63 (s, 1H, Fur), 7.13 (s, 1H, CH-Ar), 8.32 (s, 2H, NH_2_), 10.10 (s, 1H, C-6 pyrimidine), 7.42 (d, 2Н, *J* = 8.1 Hz, Ar), 7.56 (d, 2Н, *J* = 8.1 Hz, Ar), 7.52–7.64 (m, 4H, Ar). ^13^C-NMR (100 MHz, CDCl_3_) δ 105.2, 111.9, 113.8, 115.6, 116.3, 120.8, 125.7, 127.0, 127.3, 131.3, 133.3, 136.0, 136.4, 137.5, 140.0, 157.7 (C-4 pyrimidine), 160.2 (C-OH), 163.2 (C-2 pyrimidine), 169.6 (C=O), Anal. calcd. for C_21_H_14_BrN_3_O_3_: C 57.82, H 3.23, N 9.63; found: C 58.16, H 3.54, N 9.96.

### 3.3. Synthesis of Compounds ***3a**–**d***

A mixture of 0.01 moles of 5-*R*-furan-2(3*H*)-one (**1a**–**d**) and 0.015 moles of thiourea was refluxed in 20 mL of propanol-2 for 3 h, in the presence of 0.001 moles of sodium methoxide. The cooled mixture was treated with concentrated hydrochloric acid to achieve a neutral pH. The precipitated crystals were filtered off, and then washed with water. The resulting crystals were recrystallized from propanol-2.

*6-(3-Nitrophenyl)-5-[2-oxo-2-(p-tolyl)ethyl]-2-thioxotetrahydropyrimidin-4(1H)-one* (**3a**). Yield: 73%. m.p.: 168–170 °C. ^1^H-NMR (400 MHz, CDCl_3_) δ 2.05 (s, 1H, NH), 2.35 (s, 3H, CH_3_), 2.63 (dd, 1H, *J* = 14.1, 7.4 Hz, CH_2_), 3.13 (dd, 1H, *J* = 14.1, 5.7 Hz, CH_2_), 4.10 (m, 1H, CH), 4.95 (d, 1H, CH), 7.23 (d, 2Н, *J* = 8.1 Hz, *p*-Tol), 7.68 (d, 2Н, *J* = 8.1 Hz, *p*-Tol), 7.52–7.94 (m, 4H, Ar), 8.12 (s, 1H, NH). ^13^C-NMR (100 MHz, CDCl_3_) δ 28.6, 43.9, 49.2, 61.5, 118.2, 121.7, 126.5, 127.7, 129.7, 136.2, 139.4, 142.5, 145.6, 150.3, 179.4 (C=O), 187.4 (C=S), 198.6 (C=O), Anal. calcd. for C_19_H_17_N_3_O_4_S: C 59.52, H 4.47, N 10.96, S 8.36; found: C 59.85, H 4.65, N 11.32, S 8.84.

*6-(2-Chlorophenyl)-5-[2-oxo-2-(p-tolyl)ethyl]-2-thioxotetrahydropyrimidin-4(1H)-one* (**3b**). Yield: 78%. m.p.: 177–179 °C. ^1^H-NMR (400 MHz, CDCl_3_) δ 1.98 (s, 1H, NH), 2.38 (s, 3H, CH_3_), 2.69 (dd, 1H, *J* = 14.1, 7.4 Hz, CH_2_), 3.18 (dd, 1H, *J* = 14.1, 5.7 Hz, CH_2_), 4.03 (m, 1H, CH), 5.12 (d, 1H, CH), 7.28 (d, 2Н, *J* = 8.1 Hz, *p*-Tol), 7.73 (d, 2Н, *J* = 8.1 Hz, *p*-Tol), 7.18–7.43 (m, 4H, Ar), 8.20 (s, 1H, NH). ^13^C-NMR (100 MHz, CDCl_3_) δ 25.6, 42.1, 48.4, 60.0, 118.0, 120.2, 124.4, 126.2, 128.3, 134.2, 137.7 144.71, 147.6, 152.5, 177.1 (C=O), 186.3 (C=S), 201.3 (C=O), Anal. calcd. for C_19_H_17_ClN_2_O_2_S: C 61.20, H 4.60, N 7.51, S 8.60; found: C 60.96, H 4.24, N 7.89, S 9.03.

*5-[2-(4-Methoxyphenyl)-2-oxoethyl]-6-(3-nitrophenyl)-2-thioxotetrahydro-pyrimidin-4(1H)-one* (**3c**). Yield: 79%. m.p.: 189–191 °C. ^1^H-NMR (400 MHz, CDCl_3_) δ 2.10 (s, 1H, NH), 2.41 (dd, 1H, *J* = 14.1, 7.4 Hz, CH_2_), 3.09 (dd, 1H, *J* = 14.1, 5.7 Hz, CH_2_), 3.81 (s, 3H, OCH_3_), 4.07 (m, 1H, CH), 5.00 (d, 1H, CH), 7.14 (d, 2Н, *J* = 8.1 Hz, Ar), 7.45 (d, 2Н, *J* = 8.1 Hz, Ar), 7.29–7.45 (m, 4H, Ar), 8.26 (s, 1H, NH). ^13^C-NMR (100 MHz, CDCl_3_) δ 42.2, 45.3, 52.4, 57.5 121.2, 124.6, 126.0, 127.7, 130.2, 132.8, 137.3, 144.8, 151.2, 156.7, 178.0 (C=O), 189.8 (C=S), 197.7 (C=O), Anal. calcd. for C_19_H_17_N_3_O_5_S: C 57.13, H 4.29, N 10.52, S 8.03; found: C 56.87, H 3.95, N 10.93, S 8.47.

*6-(2-Chlorophenyl)-5-[2-(4-methoxyphenyl)-2-oxoethyl]-2-thioxotetrahydro-pyrimidin-4(1H)-one* (**3d**). Yield: 80%. m.p.: 181–183 °C. ^1^H-NMR (400 MHz, CDCl_3_) δ 2.13 (s, 1H, NH), 2.45 (dd, 1H, *J* = 14.1, 7.4 Hz, CH_2_), 3.13 (dd, 1H, *J* = 14.1, 5.7 Hz, CH_2_), 3.75 (s, 3H, OCH_3_), 4.12 (m, 1H, CH), 5.07 (d, 1H, CH), 7.05 (d, 2Н, *J* = 8.1 Hz, Ar), 7.69 (d, 2Н, *J* = 8.1 Hz, Ar), 7.25–7.39 (m, 4H, Ar), 8.34 (s, 1H, NH). ^13^C-NMR (100 MHz, CDCl_3_) δ 40.5, 44.6, 54.64, 59.7, 114.9, 121.7, 123.4, 125.8, 129.2, 132.5, 138.3, 142.2, 150.2, 157.2, 175.3 (C=O), 184.3 (C=S), 200.5 (C=O), Anal. calcd. for C_19_H_17_ClN_2_O_3_S: C 58.68, H 4.41, N 7.20, S 8.25; found: C 59.06, H 4.65, N 6.96, S 7.95.
